# Enhanced Spectroscopic Insight into Acceptor-Modified Barium Strontium Titanate Thin Films Deposited via the Sol–Gel Method

**DOI:** 10.3390/ma17112491

**Published:** 2024-05-22

**Authors:** Dionizy Czekaj, Agata Lisińska-Czekaj

**Affiliations:** Faculty of Mechanical Engineering and Ship Technology, Gdańsk University of Technology, 11/12, Narutowicza St., 80-233 Gdańsk, Poland; dionizy.czekaj@pg.edu.pl

**Keywords:** dielectric materials, composite thin films, impedance spectroscopy, equivalent circuit method

## Abstract

In the present paper, composite thin films of barium strontium titanate (Ba_x_Sr_1−x_TiO_3_) with an acceptor modifier (magnesium oxide—MgO) were deposited on metal substrates (stainless steel type) using the sol–gel method. The composite thin films feature Ba_x_Sr_1−x_TiO_3_ ferroelectric solid solution as the matrix and MgO linear dielectric as the reinforcement, with MgO concentrations ranging from 1 to 5 mol%. Following thermal treatment at 650 °C, the films were analyzed for their impedance response. Experimental impedance spectra were modeled using the Kohlrausch–Williams–Watts function, revealing stretching parameters (β) in the range of approximately 0.78 to 0.89 and 0.56 to 0.90 for impedance and electric modulus formalisms, respectively. Notably, films modified with 3 mol% MgO exhibited the least stretched relaxation function. Employing the electric equivalent circuit method for data analysis, the “circle fit” analysis demonstrated an increase in capacitance from 2.97 × 10^−12^ F to 5.78 × 10^−10^ F with the incorporation of 3 mol% MgO into BST-based thin films. Further analysis based on Voigt, Maxwell, and ladder circuits revealed trends in resistance and capacitance components with varying MgO contents, suggesting non-Debye-type relaxation phenomena across all tested samples.

## 1. Introduction

The history of electroceramics traces its roots to the pivotal discovery of barium titanate (BaTiO_3_), a breakthrough that reshaped our understanding of ceramics’ properties, applications, and technological possibilities. The identification of ferroelectric properties in barium titanate in 1942 marked the initiation of the modern era of ceramic dielectric materials [1]. In addition to its applications as a fundamental dielectric material, barium titanate has also been explored for its unusual semiconductor properties [2,3], exhibiting a unique positive temperature coefficient of resistance [4,5].

The piezoelectric properties of barium titanate played a crucial role in the development of ultrasonic transducers, opening up new avenues for the application of piezoelectric ceramics [6]. Due to the advantages of environmental friendliness, structural stability and high electromechanical conversion efficiency, barium titanate is one of the mainstream and sustainable piezoelectric materials for developing clean energy harvesting technologies [7].

The piezoelectric generation of ferroelectric BaTiO_3_ thin films on flexible substrates has been applied to convert mechanical energy into electrical energy [8]. Metal-insulator (BaTiO_3_)-metal-structured ribbons were transferred onto flexible substrates. The results showed that such flexible piezoelectric nanogenerators (PENGs) could power self-charging wearable electronic devices through mechanical agitations.

Research on BaTiO_3_-based nanogenerators is an important and promising area. For instance, a novel flexible nanogenerator was built by coating ITO/PET layers with a ZnO/BaTiO_3_ heterojunction composite [9]. It was found that the electrical output performance was enhanced due to the nanonetwork constructed by BaTiO_3_ microspheres and ZnO nanorods.

Polymeric matrix composites are scalable and environmentally friendly solutions to applications in harvesting mechanical energy. The use of piezoelectric polymers such as polyvinylidene fluoride (PVDF) and nano- or microparticles of rigid crystalline lead-free piezoelectric materials such as BaTiO_3_ has made it possible to fabricate flexible composite materials for piezoelectric nanogenerators (PENGs) [10].

When added to PVDF as a filler, BaTiO_3_ enhances the piezoelectric coefficient while maintaining the flexibility of the composite. The addition of BaTiO_3_ dramatically improves the electric field within the composite, leading to major improvements in the energy harvesting capabilities of the composite, specially at low BaTiO_3_ concentrations [11].

The literature survey indicates the extensive use of BaTiO_3_ as a PENG material for harvesting mechanical energy. Notable achievements include the preparation of multilayered BaTiO_3_/PVDF nanogenerators with 20 wt% BaTiO_3_ nanoparticles [12], a tailored structure by sandwiching a graphene layer between BaTiO_3_ and PVDF to enhance negative charges and improve dipole alignment [13] and a flexible PENG using Ag nanowires as the conductive filler and BaTiO_3_ as the inorganic piezoelectric material in a PVDF matrix [14].

Additionally, polymethylmethacrylate-coated BaTiO_3_ nanowires were incorporated into PVDF nanofibers [15], and polydopamine-modified BaTiO_3_/PVDF fibers were used as flexible PENGs [16]. A composite consisting of 10 wt% dopamine-modified 0.5(Ba_0.7_Ca_0.3_)TiO_3_-0.5Ba(Zr_0.2_Ti_0.8_)O_3_/PVDF showed promise as a candidate for simultaneous energy storage and harvesting by integrating into a single device [17].

Beyond its role in electronics, energy harvesting, energy storage and biomedicine (e.g., [18,19]), barium titanate and its solid solutions present promising prospects as novel photocatalysts for efficiently removing hazardous organic pollutants from water and wastewater [20]. Recent developments have also extended their use in energy and environmental protection [21].

In thin film form, ferroelectrics have three main domains of applications: namely, various sensor and actuator applications, memories and high-frequency electrical components [22]. Interest in the problem of using ferroelectric materials in microwave engineering is governed by high dielectric nonlinearity, low losses, fast switching time, low power consumption and the possibility to work under a high level of operating power [23]. The physical phenomenon that can ensure the design of, for instance, phase shifters is based on the dielectric nonlinearity of ferroelectrics [24]. Barium strontium titanate, represented as Ba_x_Sr_1−x_TiO_3_ (BST), has emerged as a noteworthy candidate for application in tunable electronically controllable microwave devices such as phase shifters, capacitors, oscillators, filters, delay lines and parametric amplifiers [6]. Despite the significant attention and potential applications, several challenges persist in the utilization of BST thin films, particularly in high-frequency scenarios [25]. In this connection, it is worth noting that one of the main challenges is the concurrent reduction of dielectric loss and augmentation of dielectric tunability.

The most interesting materials for tunable ferroelectric applications are the paraelectric phases of displacive ferroelectrics (or incipient ferroelectrics) due to their high values of dielectric permittivity and tunability even far above the Curie temperature, reduced sensitivity of the permittivity to temperature changes and the low loss level caused by the absence of ferroelectric domains [26]. The Curie point of Ba_x_Sr_1−x_TiO_3_ can be controlled by varying the amount of Sr substitution, and the material can be brought into the paraelectric phase at room temperature. In the paraelectric phase, there is no spontaneous polarization and, hence, no fatigue problem, and the dielectric properties can be tuned by an applied DC electric field. That is why paraelectric BST finds extensive applications in tunable microwave devices.

Apart from variations of the dielectric constant with the applied field, a lower value of the loss tangent (the loss factor dissipates or absorbs the incident microwave energy) is one of the most favorable properties for BST application in microwave tunable devices. To decrease the electrical losses (and dielectric permittivity) of ferroelectric barium strontium titanate, efforts include adding minor amounts of acceptor dopant ions (such as Ni^2+^, Fe^2+^, Fe^3+^, Mn^2+^, Mn^3+^, Co^2+^, Co^3+^, Cr^3+^, Bi^3+^, etc., [6]). Other endeavors involve creating composites encompassing a ferroelectric substance and the so-called linear dielectric—recognized for its minimal dielectric losses and low permittivity—such as MgO [27,28], Al_2_O_3_ [29] and SiO_2_ [25,30].

The suitable ionic radii of Mg^2+^ (0.72 Å) make it easy to diffuse into the lattice of BST thin films to substitute the Ti^4+^ (0.61 Å) ions on the B-site of the (A^2+^B^4+^O_3_^2−^) structure. By using small concentrations of acceptor ions such as Mg^2+^, the dielectric loss of the BST material can be lowered [31,32]. The dielectric loss is mainly controlled by mobile electrons. The main advantage of incorporating Mg into barium titanate is to prevent the reduction of Ti^4+^ to Ti^3+^ by neutralizing the donor action of oxygen vacancies, thus helping to lower the loss tangent and decreasing the dielectric loss [33].

The most important lead-free ferroelectrics crystallize in the perovskite structure, and many of them possess excellent properties under specific conditions. For example, barium titanate-based materials have exceptionally high piezoelectric coefficients at room temperature but depolarize at relatively low temperatures (~120 °C) [34]. On the other hand, potassium niobates (KNbO_3_) have high Curie temperatures (*T*_C_). In this regard, it should be noted that, in the case of the KNbO_3_ single crystal, the structural phase transitions from a cubic to tetragonal structure are clearly visible at 420 °C and 410 °C during heating and cooling, respectively [35,36]. In the case of KNbO_3_ ceramics, the cubic-to-tetragonal structural phase transition was found at approximately 398 °C [37]. Interest in KNbO_3_ has been renewed by a recent proposal on its potential as a base material for both nanogenerators [38] and photovoltaic applications [39]. However, the fabrication of KNbO_3_ ceramics is difficult. Among the fabrication difficulties, one can mention unsuccessful densification caused by low melting temperatures (<1100 °C), the presence of the residual K_4_Nb_6_O_17_ phase and the substantial influence of moisture on the structural stability of KNbO_3_ ceramics [37].

The main advantage of barium and strontium titanate (BST) solid solutions in thin-film form, over other lead-free ferroelectric compounds (e.g., KNbO_3_ [36], ZnO [40], AlScN [41,42], etc.), is that Ba_x_Sr_1−x_TiO_3_ exhibits a dielectric permittivity ranging from 150 to 6000 when the x parameter changes from 0 to 0.6 while the material remains in the paraelectric state at room temperature [23]. Solid solutions and doping studies on KNbO_3_ revealed that the Curie temperature decreases by approximately 15 °C for 6 mol% AgNbO_3_. The lowest Curie temperature observed in the KNbO_3_-PbTiO_3_ system was 175 °C for a composition with 20% PbTiO_3_. For the KNbO_3_-BaTiO_3_ system, the temperature for the structural phase transitions of KNbO_3_ decreases rapidly with the incorporation of BaTiO_3_ [35,43]. For instance, the dielectric constant as a function of temperature in the solid solution system of (100 − x)BaTiO_3_ + xKNbO_3_ for x values of 96 and 90 shows the cubic–tetragonal transition characterized with the maximum temperature of the dielectric constant at approximately 325 °C and 50 °C, respectively. For the system BaTiO_3_-KNbO_3_, a rather wide minimum of the Curie temperatures extends from about 35 to 75 mol% KNbO_3_ as a result of the occurrence of the complex region [35,43].

It is also worth mentioning that ferroelectricity in undoped ZnO is not a likely physical phenomenon. Only a few papers were found to claim ferroelectricity in undoped ZnO. ZnO nanostructures doped by metal ions, such as Li^+^, Mg^2+^, Co^2+^, V^5+^, Cr^3+^, Cu^2+^, Ba^2+^, Ni^2+^, K^+^, Ce^3+^, Eu^3+^, La^3+^, Gd^3+^, Nd^3+^, Y^3+^, Ho^3+^, Sm^3+^ and Dy^3+^, exhibit a dielectric anomaly (ferro-to paraelectric phase transition) and display a nonlinear hysteretic trace (ferroelectric hysteresis loop) with non-zero switchable polarization; i.e., they are ferroelectric in nature (though feeble) [40]. In the case of AlN, the addition of rare-earth metal elements into a III-nitride lattice, such as scandium (Sc) in AlN, transforms conventional III-nitrides into ferroelectrics [41,44].

One can see from the above mentioned that, even 80 years after its discovery, barium titanate and its solid solutions remain the most important engineering materials. Various techniques have been developed for the growth of BST thin films, including radio frequency (RF) magnetron sputtering [45], pulsed laser ablation deposition (PLAD) [46], DC microarc oxidation [47] and chemical vapor deposition (MOCVD). In addition to vapor deposition methods, chemical solution deposition (CSD) has been employed [48,49]. Common solution preparation methods include metal organic decomposition (MOD) and the sol–gel method [50]. Compared with the conventional synthesis methods, the sol–gel process is considered a form of nanostructure manipulation [51]. The noteworthy aspect is that the sol–gel process initiates at the nanometer scale, involving molecules, and undergoes reactions at the same level, resulting in a material with nanometer-scale characteristics [52]. Among the advantages of the sol–gel technique, one can mention the high interdiffusion of cations, control of stoichiometry at the molecular level, low synthesis temperature and small particle size [53].

An essential process widely employed in numerous domains of modern advanced technology involves depositing thin films of ferroelectric materials onto diverse substrates. For ferroelectric devices currently employed in the electronics industry, silicon (Si) stands out as the preferred substrate. Previous efforts have aimed to grow BST thin films on various substrates, including single-crystal silicon, platinum-coated silicon, Pt/Ti/SiO_2_/Si substrates and perovskite structure substrates [32,54]. There are a number of studies describing the influence of the deposition temperature on the properties of oriented BST films grown on lanthanum aluminate, magnesium oxide and sapphire orienting sublayers [55]; Pt/Ti-buffered sapphire substrate [56]; semi-insulating silicon carbide [57] or diamond substrate with a SiC buffer layer [58].

However, silicon and glass substrates, despite their use in these deposition attempts, do not meet the necessary strength requirements for materials intended for electromechanical devices like actuators or energy harvesting devices, and their use can lead to failures due to brittleness. Moreover, if ferroelectrics were to find application as integrated sensors in aerospace contexts, the substrate would need to be an integral part of a structural component, often composed of metal. Integrating functionality into structural materials, primarily metals and their alloys, holds potential for the real-time health monitoring of structural components in constructions and devices, thereby extending maintenance cycles. Furthermore, this integration opens up avenues for developing new structural functional systems [59,60]. It is clear that steel substrates offer economic benefits. However, when stainless steel is used as a substrate, lattice misfit strain, thermal strain and mutual diffusion between films and substrates can arise. These factors contribute to the development of numerous imperfections and pose challenges for the crystalline expansion and dielectric properties of thin coatings [61]. Hence, considering the practical requirements of industrial applications, it appears highly advisable to develop technology for growing efficient thin ferroelectric films on metallic substrates [61,62].

The primary objective of this investigation was to assess how the introduction of a minor amount of MgO addition (ranging from 0 to 5 mol%) affects the impedance characteristics of (Ba_0.6_Sr_0.4_)TiO_3_ thin films infused with Mg. Furthermore, a key objective of the study was to illustrate the feasibility of employing various methods for the analysis, simulation and modeling of experimental impedance spectra. The ultimate aim was to provide a comprehensive explanation of the fit results from a physical perspective.

What is new in our experiments is the use of advanced methods for modeling experimental impedance spectra recorded for BST ferroelectric thin films in the paraelectric state at room temperature. The BST thin films were grown on stainless steel substrates. By combining factors such as the composition of the BST solid solution, the type of acceptor dopant used and the selection of the substrate material, conditions were created to reduce dielectric losses, adjust the dielectric permittivity value and enable the functional integration of thin ferroelectric films with construction materials such as steel.

Particular attention was paid to the use of a convenient representation of the Kohlrausch–Williams–Watts (KWW) function [63,64] in the frequency domain [65]. The methods used for the analysis and simulation of immittance spectra included the combined utilization of complex impedance and modulus spectroscopy [66] and the electrical equivalent circuit method [67].

## 2. Materials and Methods

### 2.1. Material Synthesis

Thin films of barium strontium titanate (BST) electroceramic were applied to polished stainless steel substrates through the sol–gel spin coating technique. Spin coating is a widely used method for producing uniform and homogeneous thin films on substrates by leveraging centripetal force and surface tension. In this technique, a small volume of solution containing the coating material is dispensed onto the center of the substrate. The substrate is then rapidly rotated for a brief duration (typically a few seconds) to evenly distribute the coating material across its surface [68]. The key advantage of spin coating lies in its ability to effortlessly and rapidly produce uniform thin films of varying thicknesses from a few nanometers to several microns thick [69].

In addition to its advantageous features such as ease of operation, quick processing time (achieved through high spin speeds leading to rapid drying), consistent production of uniform, thin films and the ability to adjust the coating thickness, spin coating offers the possibility of automation for depositing multiple layers, such as functionally graded structures [70,71], making it a highly cost-effective option compared to alternative methods. However, spin coating is hindered by its reliance on a single substrate and significant solution wastage (approximately 80–90%) during the process [68]. Furthermore, it often results in uneven film thickness across the surface, compromising the overall uniformity of the final product, and is not suitable for solutions with high viscosity. Additionally, spin coating is inconsistent when dealing with large area samples [72].

Barium acetate (Ba(CH_3_COO)_2_, Sigma-Aldrich, Darmstadt, Germany, 99%), strontium acetate (Sr(CH_3_COO)_2_, Sigma-Aldrich, 99%) and tetra-butyl titanate (Ti(OC_4_H_9_)_4_, Sigma-Aldrich, 97%) served as sources of the metal elements for preparation of the (Ba_0.6_Sr_0.4_)TiO_3_ solution. Magnesium acetate (Mg(C_2_H_3_CO_2_)_2_) was used as the source of magnesium. Glacial acetic acid (CH_3_COOH) functioned as the catalyst, while *n*-butanol (CH_3_(CH_2_)_3_OH) acted as the solvent. Acetylacetone (CH_3_COCH_2_COCH_3_) was introduced as a stabilizing agent, and water was included to initiate a hydrolysis reaction. It is important to note that all the aforementioned reagents exhibited analytical purity.

Once complete dissolution was achieved in a stoichiometric manner, the precursor solution was thoroughly mixed and stirred before being applied to polished AISI 304 stainless steel substrates using the spin coating technique. Spin coating was performed at 3500 rpm for 30 s using a KW-4-type spin coater from Chemat Technology Inc., Brussels, Belgium. After coating, the films were heat-treated at 150 °C for 5 min to evaporate the solvent and volatile products. Then, the films were pyrolyzed at *T* = 350 °C for *t* = 5 min. The coating process was repeated up to 30 times, resulting in a film thickness of 600 nm [73].

The final crystallization of the freshly applied BST thin films took place in a standard environment at a temperature of *T* = 650 °C for *t* = 2 h, with a heating rate of 2 °C per minute, achieved through conventional furnace annealing.

### 2.2. Model

To facilitate electrical testing, silver electrodes were deposited on the upper surface of the thin layers using the sputtering method. This process involved employing a shadow mask to precisely define capacitors for subsequent electrical measurements. The evaluation of dielectric characteristics employed a metal–insulator–metal (MIM) capacitor arrangement. Impedance properties of the barium strontium titanate thin layers were investigated within a frequency range spanning from 10 Hz to 1 MHz under room temperature conditions, utilizing impedance spectroscopy (IS) [67,74].

The assessments were conducted using a Solartron 1260 Frequency Response Analyzer Solartron Analytical (Houston, TX, USA) and a 1296 Dielectric Interface, Solartron Analytical (Houston, TX, USA) with the amplitude of the alternating current perturbation signal set at 10 mV.

Modeling experimental impedance data involves expressing experimental data using mathematical functions or equivalent electrical circuits. A strong correspondence between the computed and observed impedance is expected while minimizing the number of parameters. It is important to highlight that, in the context of measurement modeling, the parameters frequently lack a distinct physicochemical significance.

The empirical data underwent scrutiny within the frameworks of complex impedance and complex electric modulus. Nyquist plots of complex impedance were examined using the “circle fit” method. The spectroscopic plots of both the imaginary part of impedance and the imaginary part of a complex electric modulus were modeled using the modified Kohlrausch–Williams–Watts formula. Finally, the electric equivalent circuit method was adapted to known electric equivalent circuits such as Voigt’s, Maxwell’s and ladder (or nested) circuits.

## 3. Results and Discussion

### 3.1. Impedance Spectroscopy Measurements of Sol–Gel-Derived BST-Based Composite Thin Films

Spectroscopy is the scientific study of the interactions between matter and electromagnetic radiation. This field examines phenomena such as the absorption or emission of specific types of radiation, which can be characterized by frequency, wavelength or energy. Spectroscopy plays a crucial role in various scientific disciplines, including chemistry, physics, astronomy and biology, providing valuable information about the composition, structure and behavior of matter. Different types of spectroscopy, such as UV–Visible spectroscopy, infrared spectroscopy and nuclear magnetic resonance (NMR) spectroscopy, are employed to analyze and elucidate the properties of diverse materials and substances.

Broadband dielectric spectroscopy (BBDS) refers to the field of spectroscopic research that covers the frequency range from 10^−6^ to 10^12^ Hz [75]. Coined in the late 1980s, the term is still prevalent in contemporary research. Impedance spectroscopy (IS) is a specific technique that can be considered a subset of BBDS. While broadband dielectric spectroscopy covers a wide frequency range and includes various methods for studying the dielectric properties of materials, impedance spectroscopy specifically focuses on measuring the impedance of a material as a function of frequency.

Impedance spectroscopy (IS) involves the measurement of a material’s impedance as a function of frequency. Widely applied in various fields, including electrochemistry, material science, battery research, corrosion studies and biology (e.g., [74,76]), IS is a versatile tool. Impedance, a complex quantity, characterizes the resistance a material presents to the flow of alternating current (AC) at a given frequency. It comprises two components: resistance (real part) and reactance (imaginary part). Researchers can gain valuable insights into the electrical behavior of materials and the underlying processes by measuring impedance across a range of frequencies. IS is particularly useful for analyzing systems with multiple charge transfer processes, diffusion phenomena and other dynamic behaviors. It can provide information about electrode–electrolyte interfaces, charge storage mechanisms and more [67,76].

#### 3.1.1. Bode Plot Representations of Impedance Data

**Figure 1:**
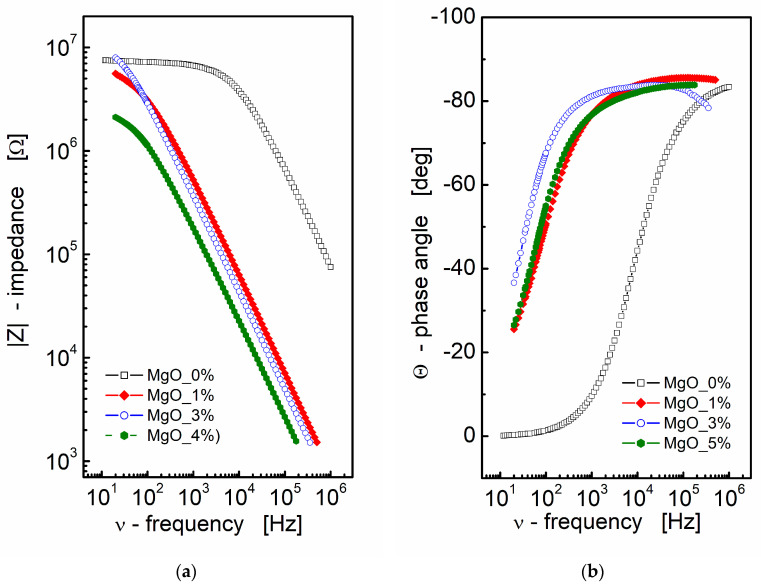
(**a**) Bode format plot of a modulus of complex impedance (|*Z*|) and (**b**) phase angle (*Θ*) vs. frequency for MgO-modified BST thin films at *T* = RT.

Impedance data can be effectively presented through Nyquist and Bode plots, graphical representations that offer valuable insights into the electrical behavior of materials or systems. In the context of BST-based composite thin films, the results of impedance spectroscopy measurements are depicted in Figure 1. A Bode format plot was chosen for the presentation, consisting of two distinct plots: one illustrating the magnitude of complex impedance as a function of frequency (Figure 1a) and another displaying the phase angle of impedance as a function of frequency (Figure 1b). It is noteworthy that the Bode format plot is recommended in this context due to the absence of frequency information in the Nyquist format, which can make it challenging to estimate initial values for fitting parameters.

From Figure 1a, it is apparent that an increase in MgO content leads to a decrease in the modulus of complex impedance across the entire frequency range for the BST-based ceramic thin films. The pure BST-based thin film (black empty squares; MgO_0; Figure 1a) exhibits a frequency-independent region (plateau) up to approximately *ν* ≈ 10 kHz. This plateau region signifies the pure resistance of the sample. As the amount of MgO additive increases, the curve representing the modulus of complex impedance shifts towards lower frequencies and smaller values of |*Z*|, indicating a decay in the “plateau region” and a shift towards a more capacitive nature in the impedance response.

Simultaneously, the slope of the dependence of the modulus of complex impedance |*Z*| on frequency remains almost constant, as observed in a log–log scale. Moving to Figure 1b, the dependence of the phase angle of the complex impedance on frequency reinforces the capacitive nature of the dielectric response in BST-based thin films modified with MgO additive. In Figure 1b, the phase angle (θ) reaches a value of approximately *Θ* ≈ −80° in the high-frequency region. This information collectively suggests that the addition of MgO induces significant changes in the dielectric properties of the BST-based thin films, altering their impedance response and capacitive behavior across different frequencies.

The analysis of the impedance data presented in Figure 1a, aided by linear fit, yielded the following results for the slope corresponding to the linear part of the dependence and the estimated values of capacitance:

- for MgO_0: slope = −0.88, C = 9.78 × 10^−12^ F,
- for MgO_1: slope = −0.93, C = 513 × 10^−12^ F,
- for MgO_3: slope = −0.93, C = 690 × 10^−12^ F,
- for MgO_5: slope = −0.90, C = 1.76 × 10^−9^ F.

These results indicate a substantial increase in the estimated value of capacitance, with an increase in the MgO content, in the BST-based thin films. The negative slope values suggest a more capacitive nature of the impedance response, and the increase in capacitance highlights the impact of MgO modification on the electrical characteristics of the thin films.

**Figure 2:**
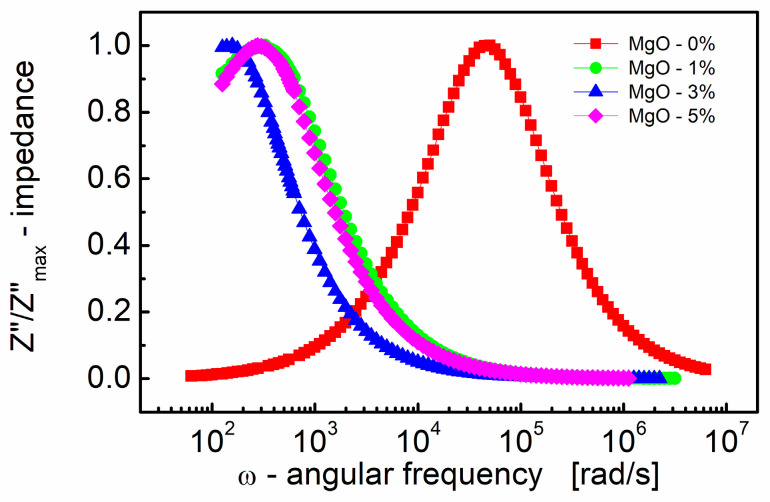
Variation of the normalized (to a peak) imaginary part of impedance (*Z*″/*Z*″_max_) with angular frequency (*ω*) for different MgO contents (semi log scale).

The choice of scaling the imaginary part of impedance and the subsequent normalization (Figure 2) has proven to be an excellent technique for revealing the relaxation mechanism within the measured frequency range. Spectroscopic plots of the normalized (to a peak) imaginary part of complex impedance (Z″/Z″_max_) are depicted in Figure 2. This representation is characterized by a peak, signifying the presence of a dielectric relaxation in the sample. The observed peak provides insights into the dynamics and behavior of the relaxation mechanism, allowing for a more detailed analysis of the dielectric response within the material.

It is well established (e.g., [77]) that peaks on the *Z*″/*Z*″_max_ spectroscopic curve are utilized to assess the relaxation frequency of the most resistive contribution, such as the electrode (~100 Hz) and/or grain boundary (~10 kHz). However, these peaks may be insufficient for minor terms, such as the bulk contribution of titanate materials with very resistive grain boundaries.

In Figure 2, it is evident that the peak is shifted to a lower frequency with the addition of even a small amount (1% by mole) of MgO. This frequency shift is notable, from *ω* ≈ 5 × 10^4^ rad/s to 300 rad/s, representing an approximate ~two orders of magnitude shift. Moreover, as the MgO content further increases, the frequency shift of the *Z*″/*Z*″_max_(*ω*) peak becomes smaller, approximately two times, compared to the MgO-1% by mole sample.

On the contrary, the position of the *Z*″/*Z*″_max_(*ω*) peak characterizes the frequency response of charge carriers. When the applied frequency approaches the frequency of electron hopping, the maxima in *Z*″/*Z*″_max_(*ω*) originate [78].

It is worth noting that the relaxation frequency of polarization mechanisms refers to the characteristic frequency it takes for the electric dipole moments of molecules or atoms to return to their original equilibrium state after being perturbed by an external electric field. Real dielectrics, however, are not perfect devices, as the resistivity of the material is not infinite and the lag or “relaxation time” of the polarization mechanisms with frequency generates losses. Ceramic dielectrics consist of atoms and ions, the latter of which largely contribute to dielectric losses. The loss contribution is maximized at the frequency where the applied field has the same period as the relaxation process (i.e., relaxation frequency = field frequency). Therefore, losses are small when the relaxation time and period of the applied field differ significantly. The practical importance of shifting the relaxation frequency by two orders of magnitude is to provide a large difference between the relaxation frequency and the field frequency, which ensures low dielectric losses during device operation.

#### 3.1.2. Modulus Formalism

The electric modulus has proven to be a valuable tool for researchers in analyzing and interpreting electrical relaxation data across diverse materials. Initially suggested and defined as the reciprocal of the complex relative permittivity [79], the concept of the electric modulus draws an analogy to mechanical shear and tensile moduli, which are complex reciprocals of shear and tensile compliances [80]. The modulus formalism proves to be particularly suitable for extracting and understanding specific phenomena such as electrode polarization and conductivity relaxation times, which are critical aspects in the study of materials, especially in the context of electrochemical and electrical properties (e.g., [81]). In essence, the modulus formalism serves as a powerful tool for dissecting and quantifying complex electrical behaviors in materials, making it instrumental in the study of various scientific and engineering applications.

The complex electric modulus (M*) is often represented using the following equation [66]:

(1) $$M^{*}=M\prime +jM\prime \prime =\frac{1}{\varepsilon^{*}}=j\omega C_{0}Z^{*}$$

Here:

- *M*′ is the real part of the complex electric modulus,
- *M*″ is the imaginary part of the complex electric modulus,
- *ω* is the angular frequency (2π*f*), where *f* is the frequency,
- *C*_0_ is the geometrical capacitance of the cell,
- Z* is the complex impedance.

**Figure 3:**
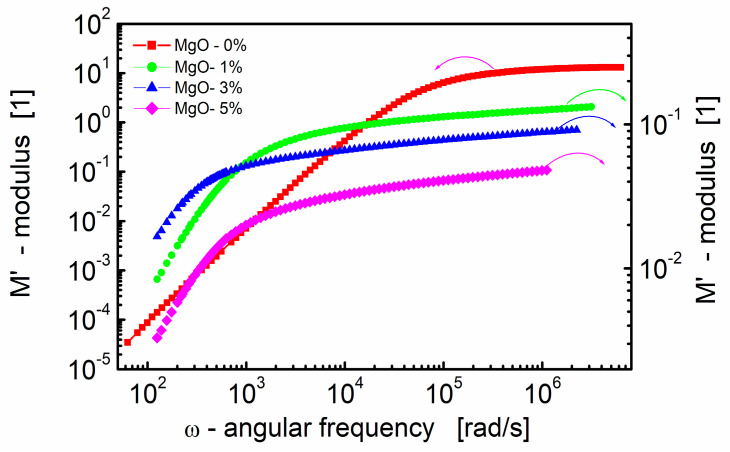
Dependence of the real part of the modulus (*M*′) on angular frequency at room temperature for the BST-based thin films with different amounts of MgO additive.

Figure 3 presents the real part of the electric modulus (*M*′) as a function of the angular frequency for BST-based thin films with varying amounts of MgO additive at room temperature. Several key observations can be made from the data.

First, at lower frequencies, *M*′ tends to be very small. This indicates that electrode effects are negligible, allowing for a more focused analysis in the modulus formalism. The impact of electrode polarization, which might be significant at low frequencies, can be ignored in this frequency range [81]. Secondly, at higher frequencies, *M*′ exhibits a constant value (*M*_∞_ = 1/*ε*_∞_). This constant value is related to the high-frequency dielectric permittivity (*ε*_∞_). Third, in the intermediate frequency range, *M*′ shows dispersion. This dispersion is indicative of relaxation processes occurring in the material. The spread of relaxation processes over a range of frequencies contributes to the observed variations in *M*′ in this frequency range.

**Figure 4:**
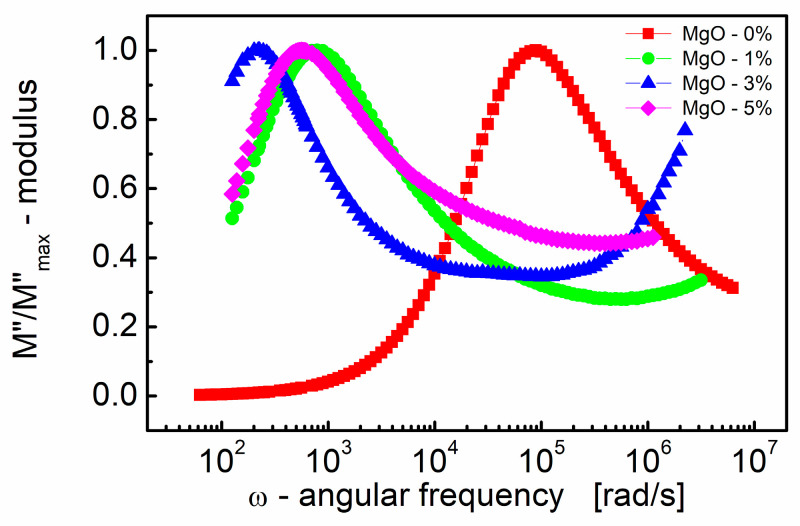
Variations of the normalized (to a peak) imaginary part of the modulus (*M*″/*M*″_max_) with angular frequency (*ω*) for different MgO contents (semi log scale).

Figure 4 illustrates how the imaginary part of the electric modulus (*M*″) varies with frequency for BST thin films with different amounts of MgO additive. One can see in Figure 4 that *M*″ exhibits a single broad relaxation peak. This peak is centered within the dispersion region of *M*′, as shown in Figure 3. The presence of a relaxation peak in the imaginary part of the electric modulus suggests the occurrence of relaxation processes within the material. The peaks observed in the *M*″ plot are broader and asymmetric on both sides of the maxima compared to the ideal Debye behavior. This asymmetry and broadening indicate that the relaxation processes in the material are more complex than a simple Debye relaxation.

The observed peak in the imaginary part of the electric modulus (*M*″) provides valuable insights into the mobility of charge carriers within the BST thin film. Here are some key points. The peak in the *M*″ plot signifies a transition in the mobility of charge carriers with the increasing frequency. Below the peak maximum, carriers exhibit long-range mobility, suggesting they can move over relatively long distances. However, as the frequency increases beyond the peak, carriers become confined to potential barriers, resulting in short-range mobility where they can only move over shorter distances.

The transition from long-range to short-range mobility indicates the existence of a hopping-type mechanism for charge transport within the material. In a hopping mechanism, charge carriers hop between localized states or sites, and the transition in mobility reflects changes in the dynamics of this hopping process [82].

The broad and asymmetric nature of the peak in the imaginary part of the electric modulus (*M*″) is attributed to the distribution of relaxation times within the material. This distribution implies that relaxation processes occur over a range of timescales, resulting in the observed broadening and asymmetry of the peak.

Combining impedance and modulus spectroscopy provides a comprehensive understanding of a material’s electric response (e.g., [66]), particularly when dealing with overlapping bulk and grain boundary arcs. Figure S1 presents combined plots of the normalized imaginary part of the modulus (*M*″/*M*″_max_) and the imaginary part of the impedance (*Z*″/*Z*″_max_) as functions of angular frequency (*ω*). The vertical lines in Figure S1 indicate the frequencies at which the curves for BST thin films modified with different amounts of MgO reach their maxima.

Peaks on spectroscopic graphs of *Z*″/*Z*″_max_(*ν*) and *M*″/*M*″_max_(*ν*) highlight different aspects of electrical behavior. The *Z*″/*Z*″_max_(*ν*) plot emphasizes phenomena with the highest resistance, while *M*″/*M*″_max_(*ν*) identifies those with the lowest capacitances [66]. The modulus representation often exhibits a peak for the bulk contribution, characterized by the lowest capacitance, typically in the order of picofarads (~1 × 10^−12^ F [83]). Conversely, the impedance representation is more suitable for assessing the relaxation frequency of the most resistant contribution [77]. In the case of titanates (e.g., [77,84]), this is often associated with grain boundaries, with a capacitance in the order of nanofarads (~1 × 10^−9^ F [83]).

Visual inspection of the modulus of the complex impedance in Figure 1a suggests that the nonmodified BST thin film exhibits relaxation polarization processes occurring at grain boundaries. The combined modulus and impedance spectroscopic plots in Figure S1 further support this observation, indicating that the relaxation frequency range is characteristic of processes occurring at grain boundaries.

### 3.2. Circle Fit of Impedance Data of Sol–Gel-Derived BST-Based Composite Thin Films

A Nyquist plot provides a graphical representation of impedance data in the complex plane, where the real part of impedance (typically representing resistance) is plotted on the *x*-axis, and the negative imaginary part of impedance (typically representing reactance) is plotted on the *y*-axis. Each point on the Nyquist plot corresponds to a specific frequency of the AC signal used in the measurement.

The shape and features of the Nyquist plot offer valuable insights into various processes occurring within a material. Characteristic patterns such as semicircles or arcs in the Nyquist plot may indicate specific electrochemical or physical phenomena, and analyzing the size and position of these features can extract information about the material’s properties. Nyquist plots are commonly used in impedance spectroscopy to understand and interpret the behavior of materials in response to alternating the current over a range of frequencies.

In an ideal scenario, the Nyquist plot exhibits a semicircle shape, which represents a combination of resistance and capacitance. This often corresponds to phenomena like electrode–(solid)electrolyte interfaces or relaxation processes within the material. However, for real materials, the Nyquist plot’s shape may deviate from the idealized semicircle. It can appear as a depressed, with the center lying below the *x*-axis, or a distorted semicircle, indicating more complex behavior. The complexity in the Nyquist plot is often referred to as non-Debye relaxation.

The Cole–Cole equation [85] is commonly used to describe non-Debye relaxation phenomena observed in impedance spectroscopy. It provides a mathematical model that accounts for the presence of multiple relaxation processes or a distribution of relaxation times within a material. The equation is expressed as [86]

(2) Z∗=ZRe+jZIm=RB+RGB1+jωRGBC1−α

Here:

- Z* is the complex impedance,
- *Z*_Re_ and *Z*_Im_ are the real and imaginary components of the impedance,
- *R_B_* is the resistance of the grain interior (bulk),
- *R_GB_* is the resistance of the grain boundary region,
- *C* represents the capacitance of the grain boundary region,
- *α* is a constant; parameter *α* is related to the depression angle *β* by the equation *α* = *β*/(π/2).

**Figure 5:**
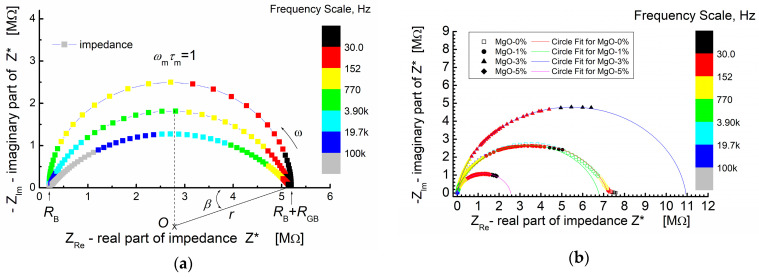
(**a**) An example of a simulated impedance plane plot for a depressed circular arc; *β*-depression angle; simulation parameters: *R*_B_ = 0.2 MΩ; *R*_GB_ = 5 MΩ; *C* = 200 pF; and *β* = 0°, 18°, and 36°. (**b**) Impedance plane plot for BST-based composite thin films modified with MgO additive and measured at *T* = RT. Symbols—denote measured impedance data points; solid lines—results of “circle fit” of the experimental data.

Figure 5a shows the simulated Nyquist plot for a system exhibiting non-Debye relaxation. The simulation parameters used were *R_B_* = 2 × 10^5^ Ω (bulk resistance), *R_GB_* = 5 × 10^6^ Ω (grain boundary resistance) and *C* = 2 × 10^−10^ F (capacitance).

Figure 5a, provides a visual comparison between an ideal semicircle and depressed semicircles (arcs) with varying angles of depression (*β*). The colored frequency scale illustrates how the position of the maximum of the arc (*ω*_m_ × *τ*_m_ = 1) shifts to higher frequencies as the depression angle increases.

In Figure 5b, the experimental impedance data points are presented by symbols, while the solid lines depict the theoretical calculations based on the “arc” model using a circle fit. This approach allows for a quantitative comparison between the theoretical model and the experimental results. The parameters obtained from the “circle fit” procedure are provided in Table 1, enabling a detailed analysis and interpretation of the complex impedance behavior observed in the study.

In Figure 5b, it is evident that the impedance spectra of the BST thin films, modified with a MgO additive and annealed at *T* = 650 °C, share some common features. The spectra are characterized by the presence of semicircles, each starting at the origin of the coordinate system (−*Z*_Im_ vs. *Z*_Re_). It is crucial to note and emphasize that the origin of the coordinate system in this representation of impedance data corresponds to the “high-frequency end” of the spectroscopic dependence of complex impedance.

In the −*Z*_Im_ vs. *Z*_Re_ representation of the impedance spectra (Figure 5b), the experimental points exhibit a monotonic increase, reaching a maximum at the point corresponding to the relaxation frequency (*ω* × *τ* = 1). Subsequently, the dependence (−*Z*_Im_ vs. *Z*_Re_) starts to decrease and smoothly tends to intersect with the real impedance value axis (*Z*_Re_). However, not all experimental curves reach this intersection with the abscissa axis. Only the impedance relationship for thin films without MgO admixture (MgO-0%, Figure 5b) achieves this intersection.

This observation suggests that the experimental “low-frequency end” of the impedance spectroscopy spectrum is “too high” to display symmetrical behavior of the relaxation function when viewed against a logarithmic frequency scale as the abscissa [65]. The diameter of the semicircle varies depending on the MgO dopant content. For low MgO content (1 mol%), the diameter of the semicircle is similar to the diameter observed for zero dopant content. The numerical parameters obtained from fitting the theoretical semicircle to the experimental impedance data of the BST-MgO thin films (using the “circle fit” method with a parallel RC circuit) are presented in Table 1.

**Figure 6:**
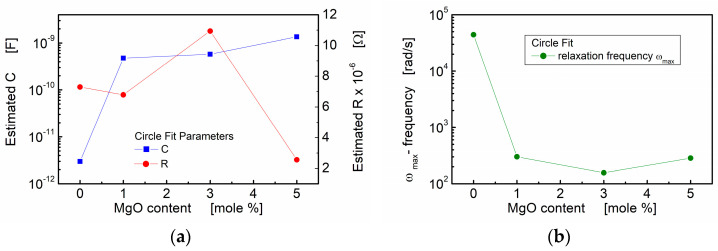
Circle fit parameters of the impedance data for BST thin films modified with a MgO additive, annealed at *T* = 650 °C: (**a**) estimated capacitance (*C*) and estimated resistance (*R*) of the grain boundary region; (**b**) estimated relaxation frequency.

In Figure 6, the influence of the MgO additive is visualized through the plotted estimated resistance (*R*), estimated capacitance (*C*) and calculated angular frequency corresponding to the relaxation processes. Notably, the estimated capacitance shows a substantial increase with an increase in MgO admixture. This increase is evident as the value rises by two to three orders of magnitude compared to the values observed for the pure BST thin film composition.

The results obtained from the “circle fit” are consistent with the “linear fit” concerning capacitance. However, the “circle fit” provides valuable additional information. The relaxation frequencies of the polarization processes were calculated and are presented in Table 1 and Figure 6b. It is noteworthy that the addition of a small amount of linear dielectric (MgO) to ferroelectric (BST) led to a significant decrease in the relaxation frequency, with a reduction of two orders of magnitude. This observation further emphasizes the influence of MgO admixture on the electrical behavior and polarization processes within the BST-MgO thin films.

### 3.3. Modeling of Impedance Data Using the Kohlrausch–Williams–Watts Function

In the analysis of the experimental spectroscopic impedance data (Figure 2), a general dielectric susceptibility function was employed, based on the postulation of power laws at high and low (limiting) frequencies [65]. This dielectric susceptibility function likely provided a framework for interpreting the impedance behavior across a wide frequency range, capturing the characteristics at both extremes of the frequency limits. Such an approach can offer a more comprehensive understanding of the dielectric response within the material under investigation.

It is noteworthy that a comparison of available phenomenological relaxation models, which are empirical relaxation functions describing the imaginary part of the dielectric susceptibility (*X*″(*ω*)), with the experimental data indicates that, when the logarithm of *X*″(*ω*) is plotted against the logarithm of frequency (log–log-scale plot), straight lines appear on both sides of a susceptibility peak. These straight lines are present at both high and low frequencies.

The slopes of these straight lines are determined by the exponents present in various relaxation models, including symmetrical ones like the widely used Debye response [87,88], the Cole–Cole equation [85] and the Fuoss–Kirkwood equation [89]. Additionally, asymmetrical empirical relaxation functions such as the Cole–Davidson function [90] and the Havriliak–Negami (HN) equation [91,92] also exhibit straight lines with slopes determined by their respective exponents.

Given the sufficiently general formulation of the formula describing the imaginary part of the susceptibility function, there was some flexibility for adapting the formula to accommodate other types of relaxations. Therefore, a three-parameter formula for relaxation in the frequency domain was applied to the analysis of the normalized amplitude (scaled) imaginary part of the impedance *Z*″/*Z*″_max_(*ω*). This formula allows for a more versatile analysis of the relaxation behavior, with the parameters offering flexibility to adapt to different relaxation processes observed in the experimental data [65]:

(3) $$\frac{Z\prime \prime }{Z{\prime \prime }_{\max}}=\frac{1}{\left(1-b\right)+\frac{b}{1+b}\left[b\left(\omega_{\max}/\omega \right)+{\left(\omega /\omega_{\max}\right)}^{b}\right]};0<b\leq 1\;$$

In Equation (3), *Z*″ represents the current value of the imaginary part of the complex impedance, *Z*″_max_ and *ω*_max_ define the height and position of the peak and “*b*” is an internally independent shape parameter for high frequencies. Notably, Equation (3) incorporates only one shape parameter (*b*) along with two parameters (*ω*_max_ and *Z*″_max_) for the peak position and height, respectively. These parameters can be easily estimated directly from spectra, providing good initial values for the curve fit routine. This simplicity enhances the applicability of the formula for curve fitting and analysis of the relaxation behavior in the material.

Equation (3) serves as a convenient representation of the Kohlrausch–Williams–Watts (KWW) function in the frequency domain [65], providing a useful frequency representation of the KWW equation. This is particularly reassuring, as the KWW equation is commonly employed to describe time domain α-relaxation data. The KWW equation describing the relaxation function is expressed as follows [63,64]:

(4) $$\phi \left(t\right)=f\exp\left[-{\left(\frac{t}{\tau }\right)}^{\beta }\right];0<\beta \leq 1\;$$

In the KWW equation (Equation (4)), *β* is the stretching parameter, and *τ* is the relaxation time. The amplitude parameter *f* is a measure of the fraction of the experimental quantity being investigated that is relaxed via the α-relaxation. A lower *β* value indicates a more stretched relaxation function *ϕ*(*t*). This equation is particularly useful for characterizing relaxation processes in the time domain, and its representation in the frequency domain (Equation (3)) allows for a convenient analysis and interpretation of the relaxation phenomena in the material under investigation. The approximate relations of the parameters of Equation (3) to the KWW parameters (Equation (4)) are employed [65]:

(5) $$b\approx \beta ,{Z\prime \prime }_{\max}\approx \frac{f}{2}\beta ,\omega_{\max}\approx \frac{1}{\tau }\frac{1}{\sqrt{\frac{1}{\beta }\Gamma \left(\frac{1}{\beta }\right)}}$$

Here, Γ is the gamma function [93].

These relationships provide a bridge between the parameters used in the analysis of the frequency domain representation (Equation (3)) and the parameters characterizing the time domain relaxation function (Equation (4)). They facilitate the interpretation and translation of findings between different domains, aiding in a more comprehensive understanding of the relaxation behavior within the material.

Equation (3) is demonstrated to be a robust frequency domain representation of the KWW equation (Equation (4)). This correspondence underscores the effectiveness of Equation (3) in capturing the essential characteristics of the relaxation phenomena described by the KWW equation. The suitability of Equation (3) for the analysis of frequency domain data further enhances its utility in elucidating the underlying relaxation processes within the BST-based electroceramic thin films.

The outcomes of modeling the normalized (in amplitude) imaginary part of impedance (*Z*″/*Z*″_max_) with the normalized frequency (*ν*/*ν*_max_) for BST thin films modified with varying contents of the MgO additive, carried out according to the function exhibiting the skewed shape given by Equation (3) (modified KWW formula), are depicted in Figure S2. The visual examination of Figure S2 reveals that the experimental data align well with the model. This representation demonstrates the effectiveness of the modified KWW formula (Equation (3)) in capturing and describing the observed frequency-dependent behavior of the imaginary part of impedance in relation to the MgO content.

The mutual alignment of the experimental points and the nonlinear fit curves confirms the high quality of the fitting procedure. Numerical indicators of the fit quality, expressed in terms of “*chi-squared*” (*χ*^2^) and “*R-squared*” (*R*^2^) parameters, are presented in Table 2. These parameters provide quantitative assessments of how well the model fits the experimental data, further supporting the validity of the modeling approach.

**Figure 7:**
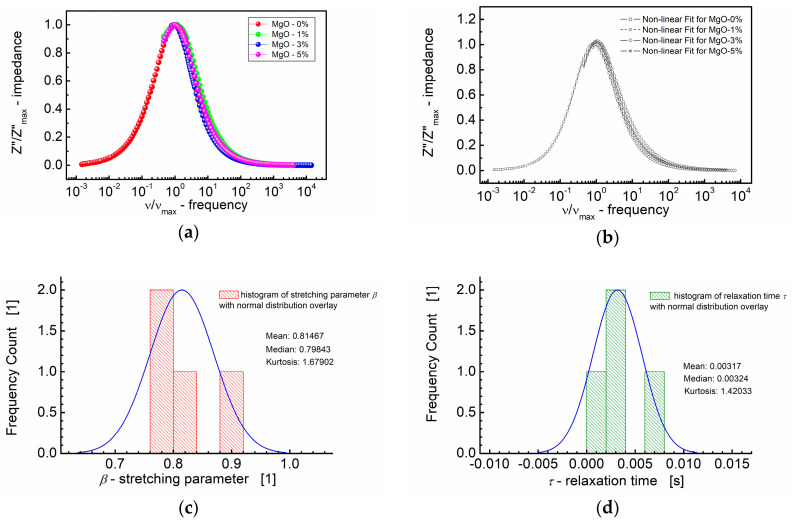
(**a**) Normalized imaginary part of impedance (*Z*″/*Z*″_max_) vs. normalized frequency (*ν/ν*_max_) for different MgO contents (symbols). (**b**) A theoretical fit of *Z*″/*Z*″_max_ vs. *ν*/*ν*_max_ performed according to the modified KWW function given by Equation (3) (semi log scale). (**c**) Histogram of the stretching parameters *β*. (**d**) Histogram of the relaxation times *τ* calculated at the base of the modified KWW function for MgO-modified BST thin films at *T* = RT.

The values of the fitting parameters “*b*” (analogous to the stretching parameter *β* in the KWW equation), amplitude parameter (*f*) and relaxation time (*τ*) are provided as functions of the MgO composition in Table 2. Notably, the maximal values for these parameters, derived from the modified KWW formula (Equation (3)), i.e., stretching parameter (*β*), amplitude parameter (*f*) and relaxation time (*τ*), were observed for the BST-based thin film with 3 mol% of MgO. Considering that a higher *β* value indicates a less stretched relaxation function (Equation (3)), it can be inferred that the relaxation function for BST thin films modified with 3 mol% of MgO additive is the least stretched among those tested. The results of these calculations are depicted in Figure 7.

Figure 7 presents a comprehensive summary, featuring both the dependence of the normalized imaginary part of impedance (*Z*″/*Z*″_max_) on the normalized frequency (*ν*/*ν*_max_) and the fitting curves obtained in accordance with the modified KWW formula given in Equation (3). This plot consolidates the key insights derived from the modeling approach, illustrating the influence of the MgO composition on the frequency-dependent behavior of the imaginary part of impedance in the BST thin films.

The curves depicted in Figure 7a,b closely align on a nearly identical “master curve”. While the general trend follows a uniform behavior, small deviations from the master curve are discernible. These deviations are captured in the distribution of the shape parameter “*b*” (Equation (3)) for high frequencies, approximating *β*, the stretching parameter of the KWW function (Figure 7c). Figure 7c illustrates that the median (≈0.80) and mean values (0.81) of the stretching parameter *β* are in close proximity. Additionally, Figure 7d presents the distribution of the relaxation times (*τ*), with mean and median values around *τ* ≈ 0.0032 s. These analyses offer insights into the statistical variations in the relaxation behavior across different MgO compositions in the BST thin films.

### 3.4. Modeling of Modulus Data Using the KWW Function

The asymmetric nature of the *M*″ plot (shown in Figure 4) suggests a stretched exponential character of the relaxation times, which can be described by the Kohlrausch–Williams–Watts (KWW) function [63,64]. Figure S3 presents the results of the modeling process of the normalized imaginary part of the modulus (*M*″/*M*″_max_) vs. normalized frequency (*ν*/*ν*_max_). Figure S3 shows how well the chosen function, derived from the KWW equation (Equation (3)), fits the experimental data for BST thin films with different MgO additive contents. The numerical indicators of the fit quality are given in Table 3.

**Figure 8:**
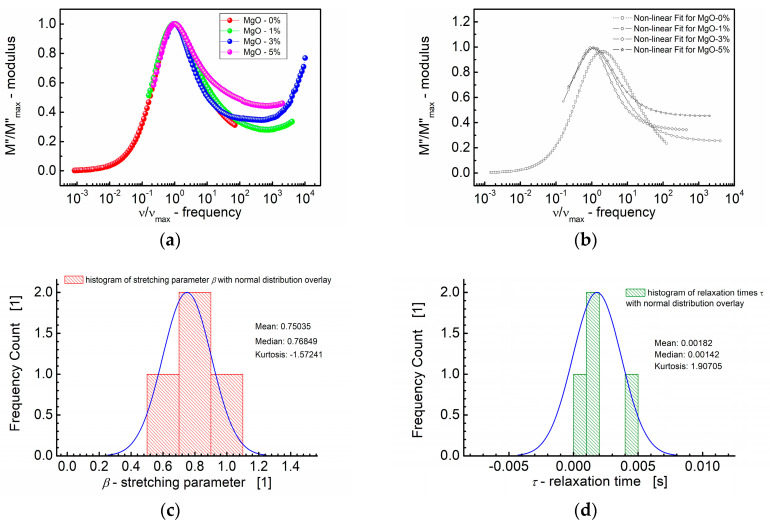
(**a**) Normalized imaginary part of the modulus (M″/M″_max_) vs. normalized frequency (*ν*/*ν*_max_) for different MgO content (symbols). (**b**) A theoretical fit according to the modified KWW function (semi log scale). (**c**) Histogram of the stretching parameters *β*. (**d**) Histogram of the relaxation times *τ* calculated at the base of the modified KWW function for MgO-modified BST thin films at *T* = RT.

The scaling behavior of the samples was studied by replotting the normalized parameters (i.e., *M*″/*M*″_max_) vs. *ν/ν*_max_; *ν*_max_ is the frequency corresponding to *M*″_max_ (Figure 8). The term “scaling behavior” implies an examination of how certain parameters change concerning one another, often with a focus on whether there is a consistent pattern or relationship that holds across different conditions or samples.

The curves depicted in Figure 8a,b show how the normalized parameters (i.e., *M*″/*M*″_max_) change concerning this characteristic frequency (*ν/ν*_max_). The significance of the modulus scaling behavior is that, on the high-frequency side, the curve represents frequencies where charge carriers are confined to their potential wells, making localized motions, whereas the low-frequency side of the peak in the *M*″/*M*″_max_ vs. *ν/ν*_max_ curve represents the frequencies at which charge carriers can move over long distances, indicating successful hopping between sites.

Based on Figure 8a,b, it can be concluded that deviations from the “master curve” are visible mostly on the high-frequency side. These deviations have been revealed by the statistical variations in the relaxation behavior across different MgO compositions in the BST thin films. First, Figure 8c illustrates the distribution of the shape parameter “*b*”, approximating the stretching parameter *β* of the KWW function. The normal distribution overlay curve was supposed, and the median (≈0.77) and mean values (0.75) of the stretching parameter *β* were derived. Furthermore, Figure 8d presents the distribution of relaxation times (*τ*), with mean and median values *τ* ≈ 0.0018 s and *τ* ≈ 0.0014 s, respectively.

### 3.5. Modeling of Impedance Data with the EEC Method

The electric equivalent circuit (EEC) method is a powerful technique used to model and analyze the behavior of complex electrical systems. It aims to represent a real-world electrical system with a simplified circuit that possesses equivalent electrical characteristics. The simplified circuit is designed to mimic the behavior of the original system under specific conditions, such as steady-state or transient conditions. Selecting an appropriate equivalent circuit model requires careful consideration and validation based on the specific characteristics of the system under investigation. One of the main challenges in equivalent circuit modeling is the fact that different equivalent models can exactly represent the same data.

**Figure 9:**
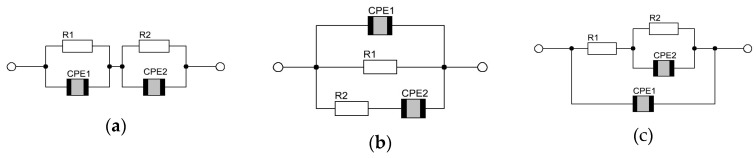
Typical circuits used in AC modeling; they are experimentally indistinguishable: (**a**) Voigt, (**b**) Maxwell and (**c**) ladder.

The most often used in measurement modeling electrical circuits are known in the literature as the Voigt, ladder (or nested) and Maxwell circuits, as presented in Figure 9 [67,76].

The Nyquist plots illustrating the impedance spectra of BST thin films, which have been modified with a MgO additive and annealed at *T* = 650 °C, are depicted in Figure 1b. It is essential to note that the impedance semicircles are displaced from the origin, with their centers positioned below the *x*-axis. Alternatively, for the estimation of the initial values for the fitting parameters, it is advisable to represent the impedance data in Bode format, indicating the magnitude of complex impedance with respect to frequency (Figure 1a) and the phase angle of impedance as a function of frequency (Figure 1b).

The outcomes of modeling the impedance spectra of BST thin films, which have been modified with varying amounts of MgO additive (0%, 1%, 3% and 5% by mole), utilizing the EEC method and three formally equivalent electrical circuits, namely Voigt, Maxwell and ladder, are presented below.

**Figure 10:**
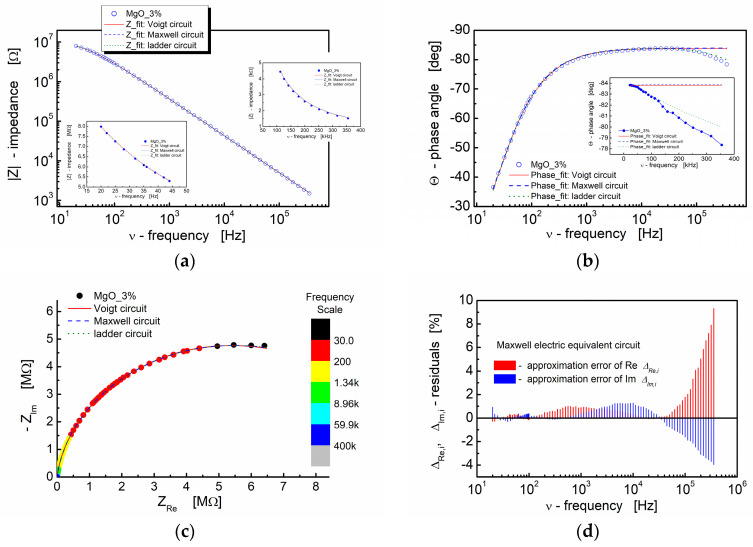
(**a**) Bode format plot of the modulus of complex impedance (|*Z*|) (log–log scale) and (**b**) phase angle (*Θ*) vs. frequency (semi-log scale) for the BST-based thin film modified with 3 mol% of MgO. (**c**) The impedance response of electrical equivalent circuits, namely Voigt, Maxwell and ladder, are shown in the figures (red solid line, blue dashed line and green dotted line, respectively). (**d**) Fit-quality plot for Maxwell electric equivalent circuit. For the graphs inserted in the main graph, a linear scale was used to visualize the differences in the curves.

The illustration of the fitting outcomes for BST thin films modified with 3% MgO additive in Figure 10 demonstrates the close correspondence between the experimental data and fitted curves, particularly in the spectroscopic trend of the modulus of the complex impedance (Bode plot, Figure 10a). All curves, encompassing both experimental and fitting ones, exhibit significant overlap. The insets in Figure 10a (linear scale) reveal that the differences are negligible at both the low-frequency and high-frequency ends of the measuring frequency range. The calculated results illustrating the spectroscopic dependence of the complex impedance phase angle, as displayed in Figure 10b, indicate slight variations in the dielectric response of the models used for fitting at the high-frequency end of the spectrum (inset in Figure 10b; linear scale).

The complex impedance plane plot (−*Z*_Im_ vs. *Z*_Re_) presented in Figure 10c demonstrates that the impedance responses of the model circuits employed for fitting align well with the experimental data. The relative deviations of the real and imaginary parts of the complex impedance (residuals) computed under the assumption of the Maxwell electric equivalent circuit are depicted in Figure 10d. It is recognized that, for a satisfactory fit, these deviations should exhibit a random distribution around the frequency axis. In our experiment, it is apparent that the residual values fall within the range of ±2% up to the frequency of *ν* = 100 kHz. For frequencies beyond 100 kHz, the approximation error for both the real and imaginary parts of the complex impedance increases. The deviation of the models from the experimental points is evident in Figure 10b for the same frequency range.

Nevertheless, as an indicator of the fitting procedure’s quality, the terms “chi-squared” (*χ*^2^) and “weighted sum of squares” (WSS) were employed [94]. In this study, “modulus data weighting” was implemented. As shown in Table 4, Table 5 and Table 6, the parameters obtained from the fitting process for the spectroscopic plots of the BST-based thin films display low values of WSS and *χ*^2^ parameters, signifying a high-quality fit.

The circuits illustrated in Figure 9 are characterized by two time constants and yield two semicircles on complex plane plots (if the time constants are “separated”). In Voigt’s model (Figure 9a), there are two parallel *R*, *CPE* subcircuits in the series. One of these subcircuits (e.g., *R*1, *CPE*1) could be interpreted as the contribution of the most resistive grain boundary, while the next one (e.g., *R*2, *CPE*2) could represent the contribution of bulk or electrode processes, including the formation of the double layer, to the total impedance of the sample. In this interpretation, the *R* and *CPE* elements constituting the circuit (Figure 9a) can be described as follows: *R*1 is the total electrical resistance of the sample (*R*_b_); *R*2 can be interpreted as the charge transfer resistance (*R*_ct_); *CPE*1 represents the constant phase element related to the geometrical capacitance of the sample (*C*_g_); *CPE*2 is the constant phase element related to the double layer capacitance (*C*_dl_) [95]. It is important to note that the Voigt circuit, in general, does not allow for the separation of the electronic and ionic components of the total electrical conductivity.

The electrical parameters of the Voigt model (Figure 9a) for BST-based thin films deposited on a stainless steel substrate are given in Table 4.

Individual, series-connected parallel *R-CPE* subcircuits represent the contribution of a specific polarization process (electrodes and contacts, grain boundaries and grain interior) to the total impedance of the sample. Here are the trends observed for the parameters.

*R*1 (total resistance of the sample): It decreases with an increase in the MgO additive up to 3% and then slightly increases. *R*2 (charge transfer resistance): It increases with an increase in the MgO additive up to 3% and then slightly decreases. *CPE*1 (geometrical capacitance of the sample): It increases with an increase in the MgO additive up to 3% and then slightly decreases. *CPE*2 (double layer capacitance; contribution of bulk or electrode processes): It increases with an increase in the MgO additive; however, for 3% additive of MgO, a small decrease occurs.

In the Maxwell’s model (Figure 9b), the interpretation of the electric parameters might be as follows: *R*1, *R*2 = the resistances related to the electronic (*R*_e_) and ionic (*R*_i_) components, respectively, *CPE*1 and *CPE*2 have the same meanings as in the Voigt’s circuit case, although the values of the parameters for both models might be different.

The electrical parameters of the Maxwell model (Figure 9b) for BST-based thin films deposited on a stainless steel substrate are given in Table 5.

The Maxwell circuit permits separation of the electronic and ionic components of the total electrical conductivity. *R*1—resistance of the electronic components—and *R*2—resistance of the ionic components—increase with an increase in the MgO additive up to 3%, then decreases. *CPE*1 (geometrical capacitance of the sample): It increases with an increase in the MgO additive.

In the nested (or ladder) circuit, the electrode/bulk interface is modeled by the double layer capacitance represented here by the constant phase element *CPE*1 connected in parallel with the Faradaic impedance. The Faradaic impedance is given by the charge transfer resistance *R*1 connected in a series with the parallel (*R*2 *CPE*2) subcircuit of a resistance and a generalized constant phase element that models the nonlinear diffusion impedance.

The electrical parameters of the ladder (nested) model (Figure 9c) for BST-based thin films deposited on a stainless steel substrate are given in Table 6.

The charge transfer resistance (*R*1) measures the change in the faradaic current as the potential across the double layer is changed while keeping the composition at the surface constant [96].

In contrast to the polarization resistance (*R*_p_ = lim*Z*(*ω*)|*ω* → 0) where the potential is changed slowly so that the surface condition changes during the perturbation, here, the potential is changed rapidly so that the surface condition has no time to change. It is the limit of the faradaic impedance at high frequencies. Therefore, it may be found from an equivalent circuit as the effective resistance in parallel with *C*_dl_ (*CPE*1, Figure 9c). For this reason, it is better to think of *R*_ct_ (*R*1, Figure 9c) as representing the dissipation of energy associated with an activation energy rather than as being specifically associated with electron transfer.

## 4. Conclusions

BST-based electroceramic thin films with (0–1) connectivity were successfully fabricated via the sol–gel method on a stainless steel substrate, incorporating Ba_0.6_Sr_0.4_TiO_3_ as the matrix and MgO as a “dispersant”. Impedance spectroscopy revealed significant effects of the MgO concentration on the electrical properties of the thin films. Different visualizations highlighted how MgO impacts the electrical characteristics (complex impedance) and dielectric (complex electric modulus) and relaxation processes.

The key observations include (i) a substantial increase in the estimated value of capacitance with the MgO content (e.g., from 2.97 × 10^−12^ F to 5.78 × 10^−10^ F as compared to the values of a pure BST thin film composition); (ii) a significant decrease in the relaxation frequency (for two orders of magnitude from 8500 Hz to 25 Hz) and (iii) the identification of non-Debye-type relaxation phenomena, as evidenced by the analysis of the stretching parameter *β* of the KWW function and the distribution of the relaxation times (*τ*). Electrical equivalent circuit analysis further supported these findings, revealing changes in the sample resistance and component capacitances with the MgO addition.

Overall, this study provides valuable insights into the influence of the MgO concentration on the electrical properties of BST-based thin films, offering potential avenues for optimizing their performance in various applications.

## Figures and Tables

**Table 1:** Parameters of the “circle fit” procedure of the BST-MgO thin film impedance data.

Circle Fit Parameter	MgO-0%	MgO-1%	MgO-3%	MgO-5%
Real Centre, [Ω]	3.5699 × 10^6^	3.3761 × 10^6^	5.4666 × 10^6^	1.2765 × 10^6^
Imaginary Centre, [Ω]	1.0952 × 10^6^	8.22 × 10^5^	7.3195 × 10^5^	2.2366 × 10^5^
Diameter, [Ω]	7.6146 × 10^6^	6.9825 × 10^6^	1.1036 × 10^7^	2.5946 × 10^6^
Deviation, [Ω]	47,334	28,750	9277.3	4703.5
Low Intercept, [Ω]	−76,471	−17,020	−2716.6	−1341.6
High Intercept, [Ω]	7.2163 × 10^6^	6.7692 × 10^6^	1.0936 × 10^7^	2.5544 × 10^6^
Depression Angle, [deg]	16.718	13.618	7.6225	9.9276
*ω*_max_, [rad/s]	44,180	301.25	156.84	284.59
Relaxation time *τ*, [s]	2.263467 × 10^−5^	0.003319	0.006375	0.003513
Estimated *R*, [Ω]	7.2927 × 10^6^	6.7862 × 10^6^	1.0939 × 10^7^	2.5558 × 10^6^
Estimated *C*, [F]	2.9726 × 10^−12^	4.754 × 10^−10^	5.7772 × 10^−10^	1.3543 × 10^−9^

**Table 2:** The value of the fitting parameters obtained by a modified KWW equation (Equation (3)) for BST-based composite thin films deposited on stainless steel. Modeling of (*Z*″/*Z*″_max_) vs. (*ν*/*ν*_max_).

Fit Parameter	MgO-0%	MgO-1%	MgO-3%	MgO-5%
Stretching parameter, *β* (or “*b*” parameter)	0.7833 ± 0.00691	0.76936 ± 0.00696	0.89248 ± 0.00203	0.81356 ± 0.00511
Amplitude parameter, *f* [Ω]	6.97654 × 10^6^	6.83641 × 10^6^	10.7322 × 10^6^	2.62313 × 10^6^
Relaxation time, *τ* [s]	1.86788 × 10^−5^	0.00307	0.00619	0.00342
“chi-squared” *χ*^2^	0.0005	0.00041	0.00001	0.00018
“R-squared” *R*^2^	0.99552	0.99767	0.99991	0.99895

**Table 3:** The values of the fitting parameters obtained by the modified KWW equation (Equation (3)) for BST-based thin films. Modeling of (*M*″/*M*″_max_) vs. (*ν*/*ν*_max_).

Fit Parameter	MgO-0%	MgO-1%	MgO-3%	MgO-5%
Stretching parameter, *β* (or “*b*” parameter)	0.56013 ± 0.00574	0.70085 ± 0.00524	0.9043 ± 0.00261	0.83614 ± 0.0142
Amplitude parameter, *f* [-]	17.366	9.769 × 10^−2^	5.438 × 10^−2^	2.703 × 10^−2^
Relaxation time, *τ* [s]	8.75599 × 10^−6^	0.00113	0.00444	0.0017
“chi-squared” *χ*^2^	0.00071	0.00022	0.00001	0.00051
“R-squared” *R*^2^	0.99397	0.99683	0.99983	0.98872

**Table 4:** Electrical parameters of the Voigt model for BST-based thin films.

Electrical and Fit Quality Parameters	Value BST-MgO-0%	Value BST-MgO-1%	Value BST-MgO-3%	Value BST-MgO-5%
R1, [Ω]	3.5502 × 10^6^	1.7558 × 10^6^	1.0877 × 10^5^	4.2916 × 10^5^
CPE1-T, [Ω^−1^ s^P^]	4.2293 × 10^−11^	8.3366 × 10^−10^	9.0551 × 10^−9^	4.4485 × 10^−9^
CPE1-P, [1]	0.88476	0.93774	1	0.91206
R2, [Ω]	3.6778 × 10^6^	4.9903 × 10^6^	1.0631 × 10^7^	2.2225 × 10^6^
CPE2-T, [Ω^−1^ s^P^]	7.2833 × 10^−12^	1.2024 × 10^−9^	8.4394 × 10^−10^	2.9591 × 10^−9^
CPE2-P, [1]	0.93795	0.9462	0.92907	0.92125
“chi-squared” *χ*^2^	0.00038978	0.00031905	0.00027061	0.00037669
WSS	0.075618	0.063171	0.052228	0.067804

**Table 5:** Electrical parameters of the Maxwell model for BST-based thin films.

Electrical and Fit Quality Parameters	Value BST-MgO-0%	Value BST-MgO-1%	Value BST-MgO-3%	Value BST-MgO-5%
R1, [Ω]	7.3769 × 10^6^	1.0526 × 10^7^	1.0994 × 10^7^	3.0928 × 10^6^
CPE1-T, [Ω^−1^ s^P^]	4.773 × 10^−12^	4.1147 × 10^−10^	7.8023 × 10^−10^	1.3999 × 10^−9^
CPE1-P, [1]	0.94414	0.9542	0.93231	0.93559
R2, [Ω]	6.2357 × 10^6^	100 (fixed)	4.3673 × 10^7^	1.5862 × 10^5^
CPE2-T, [Ω^−1^ s^P^]	1.1824 × 10^−10^	1.4119 × 10^−8^	2.0024 × 10^−10^	8.6415 × 10^−9^
CPE2-P, [1]	0.55547	0.33138	0.68523	0.5038
“chi-squared” χ^2^	0.00026467	5.1416 × 10^−5^	0.00027315	0.00016193
WSS	0.051347	0.010232	0.052445	0.029148

**Table 6:** Electrical parameters of the ladder model for BST-based thin films.

Electrical and Fit Quality Parameters	Value BST-MgO-0%	Value BST-MgO-1%	Value BST-MgO-3%	Value BST-MgO-5%
R1, [Ω]	3.3792 × 10^6^	9712 (fixed)	966.3	1.5097 × 10^5^ (fixed)
CPE1-T, [Ω^−1^ s^P^]	4.773 × 10^−12^	4.116 × 10^−10^	1.7671 × 10^−10^	1.3999 × 10^−9^
CPE1-P, [1]	0.94414	0.95418	1 (fixed)	0.93559
R2, [Ω]	3.9976 × 10^6^	1.051 × 10^7^	1.083 × 10^7^	2.9418 × 10^6^
CPE2-T, [Ω^−1^ s^P^]	4.0263 × 10^−10^	1.4118 × 10^−8^	7.4196 × 10^−10^	9.5512 × 10^−9^
CPE2-P, [1]	0.55547	0.33175	0.87912	0.50379
“chi-squared” *χ*^2^	0.00026467	5.1427 × 10^−5^	7.2684 × 10^−5^	0.00016104
WSS	0.051347	0.010234	0.014028	0.029148

## Data Availability

The data presented in this study are available on request from the corresponding author. The data are not publicly available due to privacy reason.

## Supplementary Materials

The following supporting information can be downloaded at https://www.mdpi.com/article/10.3390/ma17112491/s1, Figure S1: Normalized plots of imaginary part of electric modulus (M″/M″_max_) and impedance (Z″/Z″_max_) of MgO modified BST thin films as a function of angular frequency (*ω*) (semi log scale) for different MgO content at *T* = RT; (a) 0% by mole MgO content; (b) 1% by mole MgO content; (c) 3% by mole MgO content; (d) 5% by mole MgO content. The vertical dashed lines show the position of the maxima; Figure S2: Normalized imaginary part of impedance (Z″/Z″_max_) versus normalized frequency (ν/ν_max_) for different MgO content (symbols) and theoretical fit according to the modified KWW function given by Equation (5); (a) 0% by mole MgO content; (b) 1% by mole MgO content; (c) 3% by mole MgO content; (d) 5% by mole MgO content. Values of quality parameters (*χ*^2^ and *R*^2^) and stretching parameter *β* from KWW equation are given in the legend; Figure S3: Normalized imaginary part of modulus (M″/M″_max_) versus normalized frequency (ν/ν_max_) for different MgO content (symbols) and theoretical fit according to the modified KWW function given by Equation (5); (a) 0% by mole MgO content; (b) 1% by mole MgO content; (c) 3% by mole MgO content; (d) 5% by mole MgO content. Values of quality parameters (*χ*^2^ and *R*^2^) and stretching parameter *β* from KWW equation are given in the legend.
