# Supplementary material for: Enhanced Spectroscopic Insight into Acceptor-Modified Barium Strontium Titanate Thin Films Deposited via the Sol–Gel Method
Source: Materials (Basel). 2024 May 22;17(11):2491. doi: 10.3390/ma17112491 (PMC11172994; doi:10.3390/ma17112491)
Supplement: Supplementary file 1 [file materials-17-02491-s001.zip › materials-2849916-supplementary.pdf]

materials

Supplementary information

Enhanced Spectroscopic Insight into Acceptor-Modified Barium Strontium Titanate Thin Films Deposited via the Sol–Gel Method

Dionizy Czekaj and Agata Lisińska-Czekaj *

Faculty of Mechanical Engineering and Ship Technology, Gdańsk University of Technology, 11/12, Narutowicza St., 80-233 Gdańsk, Poland; dionizy.czekaj@pg.edu.pl
* Correspondence: agata.czekaj@pg.edu.pl

Figure S1 contains combined plots of the normalized (with respect to the amplitude) imaginary part of the modulus (M''/M''_{max}) and the imaginary part of the impedance (Z''/Z''_{max}) as functions of angular frequency (ω). The vertical lines in Figure S1 indicate the frequencies at which the curves for BST thin films modified with different amounts of MgO reach their maxima. The combined modulus and impedance spectroscopic plots in Figure S1 support observation (shown in Figure 1*a*) that the non-modified BST thin film exhibits relaxation polarization processes occurring at grain boundaries. The relaxation frequency range is characteristic of processes occurring at grain boundaries.

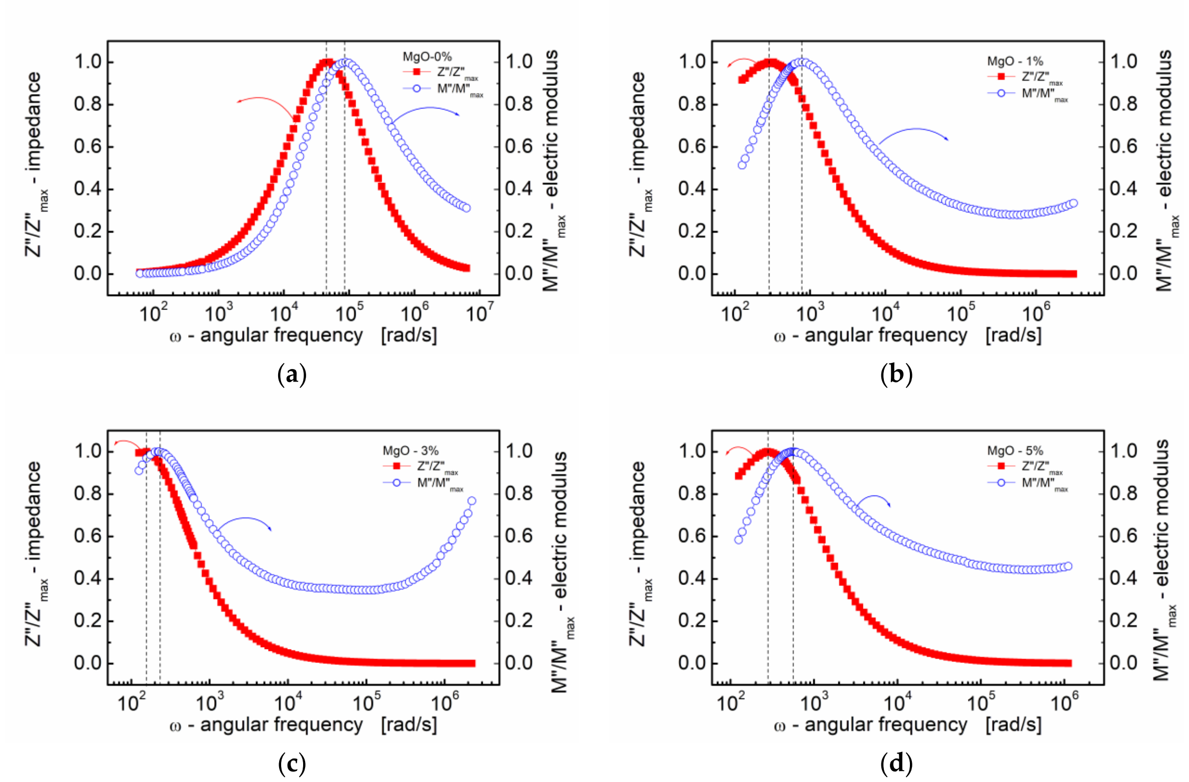

Figure S1. Normalized plots of imaginary part of electric modulus (M''/M''_{max}) and impedance (Z''/Z''_{max}) of MgO modified BST thin films as a function of angular frequency (*ω*) (*semi log scale*) for different MgO content at *T*=RT; ***a)*** 0% by mole MgO content; ***b)*** 1% by mole MgO content; ***c)*** 3% by mole MgO content; ***d)*** 5% by mole MgO content. The vertical dashed lines show the position of the maxima.

The results of modelling the imaginary part of impedance (Z''/Z''_{max}) with frequency (ν/ν_{max}) for BST thin films modified with varying content of MgO additive are depicted in Figure S2. The visual examination of Figure S2 reveals that the experimental data align well with the model. This representation demonstrates the effectiveness of the modified KWW formula in capturing and describing the observed frequency-dependent behaviour of the imaginary part of impedance in relation to MgO content.

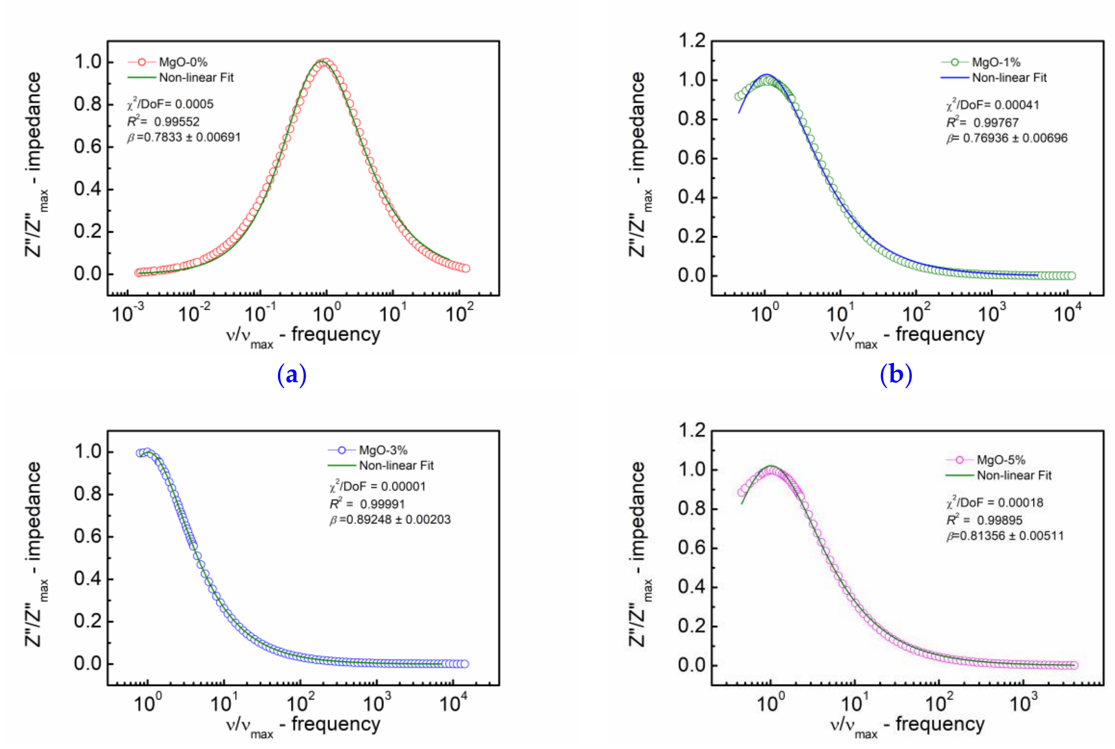

(a) (b) (c) (d)

Figure S2. Normalized imaginary part of impedance (Z''/Z''_{max}) versus normalized frequency (ν/ν_{max}) for different MgO content (symbols) and theoretical fit according to the modified KWW function given by Equation 5; ***a)*** 0% by mole MgO content ***b)*** 1% by mole MgO content ***c)*** 3% by mole MgO content ***d)*** 5% by mole MgO content. Values of quality parameters (χ^2 and R^2) and stretching parameter β from KWW equation are given in the legend.

Figure S3 presents the results of the modelling process, showing how well the chosen function, derived from the Kohlrausch-Williams-Watts KWW equation fits the experimental data for BST thin films with different MgO additive contents.

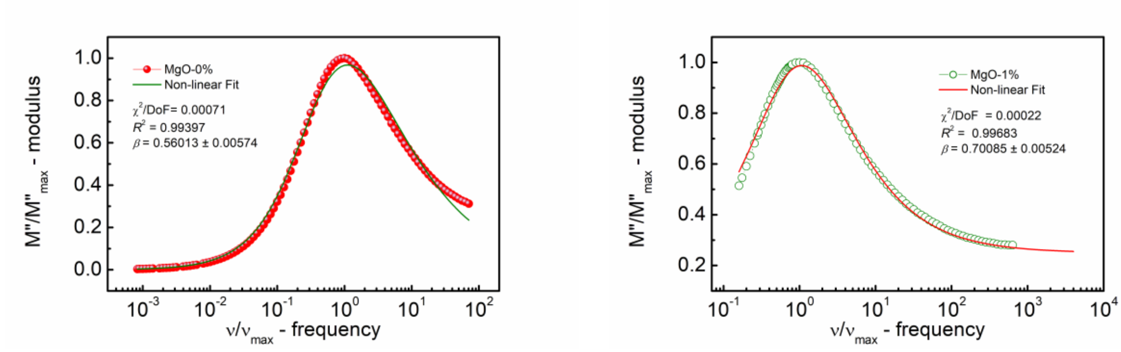

(a) (b)

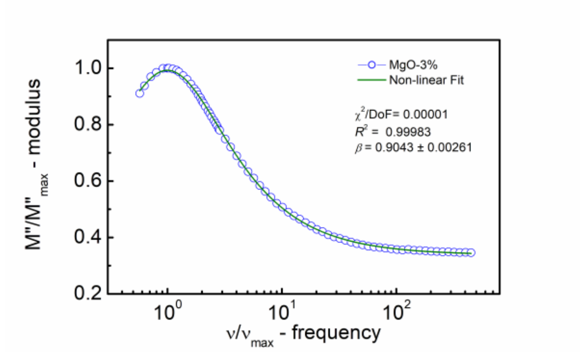

(**c**)

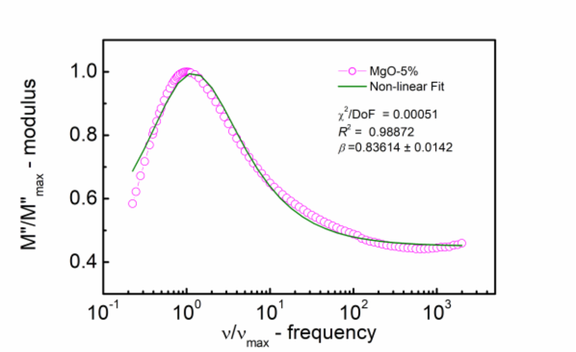

(**d**)

Figure S3. Normalized imaginary part of modulus (M''/M''_{max}) versus normalized frequency (ν/ν_{max}) for different MgO content (symbols) and theoretical fit according to the modified KWW function given by Equation 5 ; ***a)*** 0% by mole MgO content ***b)*** 1% by mole MgO content ***c)*** 3% by mole MgO content ***d)*** 5% by mole MgO content. Values of quality parameters (χ^2 and R^2) and stretching parameter β from KWW equation are given in the legend.
